# Predicting Alzheimer’s Disease Conversion From Mild Cognitive Impairment Using an Extreme Learning Machine-Based Grading Method With Multimodal Data

**DOI:** 10.3389/fnagi.2020.00077

**Published:** 2020-04-01

**Authors:** Weiming Lin, Qinquan Gao, Jiangnan Yuan, Zhiying Chen, Chenwei Feng, Weisheng Chen, Min Du, Tong Tong

**Affiliations:** ^1^School of Opto-Electronic and Communication Engineering, Xiamen University of Technology, Xiamen, China; ^2^College of Physics and Information Engineering, Fuzhou University, Fuzhou, China; ^3^Imperial Vision Technology, Fuzhou, China; ^4^Fujian Key Laboratory of Communication Network and Information Processing, Xiamen University of Technology, Xiamen, China; ^5^School of Electrical Engineering & Automation, Xiamen University of Technology, Xiamen, China; ^6^Department of Thoracic Surgery, Fujian Cancer Hospital, Fuzhou, China; ^7^Fujian Provincial Key Laboratory of Eco-Industrial Green Technology, Wuyi University, Wuyishan, China; ^8^Fujian Key Lab of Medical Instrumentation and Pharmaceutical Technology, Fuzhou University, Fuzhou, China

**Keywords:** Alzheimer’s disease, extreme learning machine, mild cognitive impairment, multimodal, prediction

## Abstract

Identifying patients with mild cognitive impairment (MCI) who are at high risk of progressing to Alzheimer’s disease (AD) is crucial for early treatment of AD. However, it is difficult to predict the cognitive states of patients. This study developed an extreme learning machine (ELM)-based grading method to efficiently fuse multimodal data and predict MCI-to-AD conversion. First, features were extracted from magnetic resonance (MR) images, and useful features were selected using a feature selection method. Second, multiple modalities of MCI subjects, including MRI, positron emission tomography, cerebrospinal fluid biomarkers, and gene data, were individually graded using the ELM method. Finally, these grading scores calculated from different modalities were fed into a classifier to discriminate subjects with progressive MCI from those with stable MCI. The proposed approach has been validated on the Alzheimer’s Disease Neuroimaging Initiative (ADNI) cohort, and an accuracy of 84.7% was achieved for an AD prediction within 3 years. Experiments on predicting AD conversion from MCI within different periods showed similar results with the 3-year prediction. The experimental results demonstrate that the proposed approach benefits from the efficient fusion of four modalities, resulting in an accurate prediction of MCI-to-AD conversion.

## Introduction

Alzheimer’s disease (AD) is the most common cognitive impairment disease, which gradually impacts the activities of a patient’s daily life. The number of AD patients was estimated to be approximately 30 million in 2015 (Vos et al., 2016), which has placed a huge socioeconomic burden on those taking care of AD patients. The pathology changes of AD begin several years before the first clinical symptoms, and mild cognitive impairment (MCI) is thought to be the prodromal stage of AD (Markesbery and Lovell, 2010). Approximately 10–17% of those with MCI progress to AD over the course of a few years, yet some MCI patients remain stable after several years (Hamel et al., 2015). It is crucial to identify people who are at high risk of progressing from MCI to AD because it can help physicians treat these patients sooner and apply suitable therapies to slow down the progression or even improve a patient’s condition. Numerous studies have used machine learning techniques for computer-aided diagnosis of AD or prediction of AD conversion. The diagnosis of AD is relatively easier than the prediction of AD because there are apparent differences between AD and a normal control (NC), and the accuracy of diagnosis has reached to above 96% (Lei et al., 2016; Kim and Lee, 2018). However, the prediction of AD, more specifically, discriminating progressive MCI (pMCI) from stable MCI (sMCI), is more challenging because the differences between these two groups are slight.

Different modalities of medical data have been used to detect the pathology associated with AD. Structural magnetic resonance imaging (sMRI) is one of the most widely used modality due to its high resolution and non-invasive characteristics (Querbes et al., 2009; Oliveira et al., 2010; Coupé et al., 2012; Eskildsen et al., 2013). AD patients are always accompanied by cerebral atrophy or ventricular expansion that is caused by the death of neurons in the affected regions. The cerebral atrophy patterns associated with AD can be revealed by MRI, and MRI is a good detection technique for the atrophy of AD. Moradi et al. (2015) calculated an MRI-based biomarker for the prediction of MCI-to-AD conversion. Tong et al. (2017a) applied an elastic net regression to grade MRI features and to predict MCI-to-AD conversion. Lin et al. (2018) used a convolutional neural network-based framework to extract high-level AD-related features from MRI for the prediction of AD. These methods only focused on MRI data and could only predict a 3-year AD conversion with an accuracy no greater than 80%. Fluorodeoxyglucose positron emission tomography (FDG-PET) is another useful neuroimaging modality for the detection of AD. Studies (Mosconi et al., 2009; JackJr., Knopman et al., 2010; Landau et al., 2011) have shown that AD and MCI patients have reduced glucose metabolism in certain cerebral regions, which occur prior to the changes in brain structure. The brain’s metabolic activity can be quantitatively measured by FDG-PET, which makes FDG-PET a potential tool for the early detection of AD (Gray et al., 2013; Cheng et al., 2015; Iaccarino et al., 2017). In a recent study (Lu et al., 2018), FDG-PET images were used in a multiscale deep neural network to classify AD/NC and pMCI/sMCI, where accuracies of 93.58 and 82.51% were achieved, respectively. In addition to MRI and FDG-PET, biological biomarkers can also contribute to the detection of AD. The abnormal concentrations of proteins in cerebrospinal fluid (CSF), such as total tau (T-tau), hyperphosphorylated tau (P-tau), and the 42 amino acid isoforms of amyloid β (Aβ42), are some of the earliest signs of AD that occur many years before the onset of clinical symptoms (Niemantsverdriet et al., 2017). Therefore, these biomarkers can provide valuable information for the early detection of AD. Genetics are also an important indicator of the risk of AD. Individuals with the apolipoprotein E (APOE) ϵ4 gene have a much higher risk of developing AD than those without APOE ϵ4 (Vounou et al., 2012; Lambert et al., 2013). Taking APOE ϵ4 into account with imaging or biological biomarkers can improve the accuracy of AD prediction.

Different modalities of biomarkers reflect the AD-related pathological changes in different aspects, thus there may be complementary information among several modalities. Combining multimodal biomarkers would provide more information and improve the accuracy of AD prediction. A simple way to fuse different modalities is to directly concatenate multimodal features and feed them into a classifier (Kohannim et al., 2010; Walhovd et al., 2010; Westman et al., 2012). However, this is not the optimal approach, and it can lead to bias of the modality with a larger number of features. A better way is to map these multimodal features into a kernel space before concatenation (Hinrichs et al., 2011; Zhang et al., 2011; Young et al., 2013), but these methods are sensitive to the weight assigned to each modality. In recent years, deep learning architecture has been employed to extract multimodal feature representations. Liu et al. (2015) used stacked auto-encoders and a zero-mask strategy to fuse MRI and PET data. Suk et al. (2014) proposed a joint feature representation of MRI and PET with a multimodal deep Boltzmann machine. Liu et al. (2018) constructed multiple deep three-dimensional (3D) convolutional neural networks to transform MRI and PET images into compact high-level features. These deep learning-based methods achieved promising results in the classification of AD/NC, but the accuracy of classifying pMCI/sMCI was just 74.58% (Suk et al., 2014). To exploit the complementarity across multimodal data, Tong et al. (2017b) employed a non-linear graph fusion that achieved better results in the diagnosis of AD and a three-way classification of AD/MCI/NC than the approaches based on a linear combination, but the classification of pMCI from sMCI was not validated. Although all of these multimodal data-based methods achieved promising results in the diagnosis of AD, the performance of AD prediction needs to be further improved for clinical use with the help of an efficient fusion of multimodal biomarkers.

Since the efficient multimodality fusion can improve the performance of an artificial intelligence system (Hu et al., 2018), in this work, we present a novel extreme learning machine (ELM)-based (Huang et al., 2012) grading method to combine four modalities (MRI, FDG-PET, CSF, and APOE ϵ4) that predict MCI-to-AD conversion. Specifically, each modality feature, from the MCI subjects, was individually graded by an ELM that trained with the corresponding modality features of AD and NC, and the grading score represented the similarity of MCI-to-AD or NC. Then, the scores of all modalities were concatenated and fed to an ELM classifier for classification of pMCI/sMCI. The results of the proposed method were evaluated by 100 runs of 10-fold cross-validation with data from the ADNI cohort. The contributions of this paper are as follows:

(i) Useful information about AD/NC was included by using the AD/NC features when training the grading ELMs, which improved the process of discriminating pMCI from sMCI.
(ii) These grading ELMs were trained with discrete labels of AD/NC and modified to output grading values, instead of discrete labels, to represent the similarity of MCI to AD or NC.
(iii) Each modality was graded into one single score, avoiding bias of the modality with a greater number of features.
(iv) The proposed approach achieved promising results in the prediction of MCI-to-AD conversion.

## Materials

The multimodal data used in this study included 313 MRI features, 20 FDG-PET features, three CSF biomarkers, and one gene feature. The MRI features, consisting of volume, surface area, and cortical thickness of the cerebral regions, were obtained through analysis with the FreeSurfer software using cross-sectional processing (Fischl and Dale, 2000; Fischl et al., 2004). There was 345 features obtained from the FreeSurfer analysis; however, because 32 features were absent from most subjects, only 313 MRI features were selected. For FDG-PET scans, five regions, frequently cited in FDG-PET studies of AD, were adopted, including left angular, right angular, bilateral posterior cingulate, left inferior temporal, and right inferior temporal (Landau et al., 2010; Landau et al., 2011). The mean, minimum, maximum, and standard deviation values of the intensity in each region were taken as the FDG-PET features. The levels of the biomarkers Aβ42, T-tau, and P-tau in CSF were used as the CSF features. The gene feature was a single categorical variable indicating the presence of APOE ϵ4 in subjects. All the multimodal data were downloaded from the ADNI website. Specifically, the MRI, CSF, and gene data were provided by the Tadpole Challenge Data files, and the FDG-PET data were provided by the UC Berkeley FDG Analysis file.

To date, there have been over 1,500 participants, ages 55 to 90 years, recruited by ADNI, and most of them were visited and tested multiple times in the following years for long-term study. In this study, we only take baseline data to predict the future state (progress to AD or remain MCI) for MCI subjects. Because not all subjects underwent all possible examinations, we excluded subjects without all modalities data available at the baseline visit, which presented 200 NC subjects, 102 AD subjects, 110 pMCI subjects who converted to AD within 3 years, and 205 sMCI subjects who did not convert to AD. Demographic and clinical information of these subjects are listed in Table 1, including gender, age, education history, and Mini Mental State Examination (MMSE) score.

**TABLE 1:** The demographic information of subjects.

**Mean ± SD**	**NC**	**sMCI**	**pMCI**	**AD**
Count (F/M)	200 (93/107)	205 (90/115)	110 (47/63)	102 (35/67)
Age	73.9 ± 6.0	71.8 ± 7.1	73.9 ± 7.2	75.7 ± 8.0
Education	16.4 ± 2.7	16.1 ± 2.7	16.2 ± 2.7	15.4 ± 3.0
MMSE	29.0 ± 1.2	28.1 ± 1.7	27.1 ± 1.7	23.2 ± 2.0

*In cells of the second row, the first number is total number with numbers of female (F) and male (M) in brackets. AD, Alzheimer’s disease; MMSE, Mini Mental State Examination; NC, normal control; pMCI, progressive mild cognitive impairment; SD, standard deviation; sMCI, stable mild cognitive impairment.*

## Methods

**FIGURE 1:**
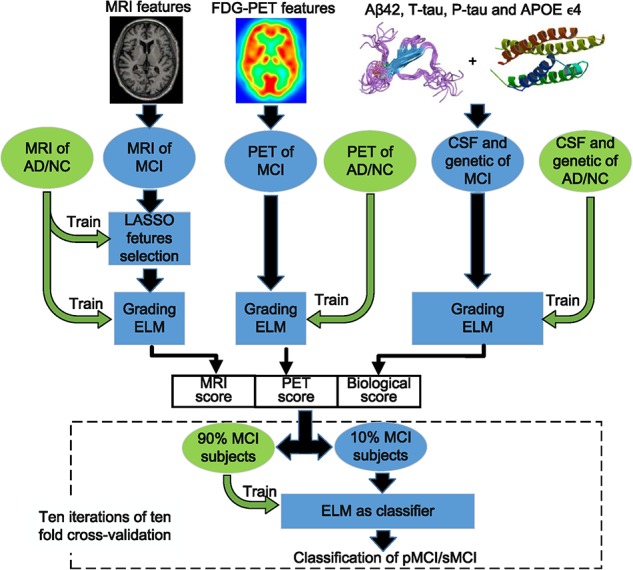
The overall framework of the proposed approach.

The overall framework of the proposed approach is shown in Figure 1, and we also summarize the process of our proposed approach as pseudo-code in Algorithm 1. There are three major steps in this framework: (i) MRI features are first preprocessed by feature selection with the least absolute shrinkage and selection operator (LASSO) algorithm; (ii) each modality (CSF and gene are combined as biological modality) of MCI is graded by ELM. These ELMs are trained with corresponding modality of features and labels from AD/NC groups. A grading score is calculated for each modality, which represents the similarity of MCI-to-AD or NC; (iii) these scores are combined to form the new representative features of MCI and fed into an ELM classifier to discriminate pMCI from sMCI. Ten-fold cross-validation is utilized to assess the performance of the proposed approach. Before these steps, all features of AD/NC are first normalized to have zero mean and unit variance. The features of MCI are also normalized with the mean and deviation of the AD/NC features. In the following sections, we will present the details of these steps.

**ALGORITHM 1:** The pseudo-code of the proposed method.

**Input: *M***_AD/NC_, ***P***_AD/NC_, ***B***_AD/NC_, ***M***_MCI_, ***P***_MCI_, ***B***_MCI_
1 **α** = LASSO(train = ***M***_AD/NC_).coefficients;
2 ***M***_AD/NC_ = ***M***_AD/NC_[:, **α**! = 0], ***M***_MCI_ = ***M***_MCI_[:, **α**! = 0];
3 score_MRI_ = ELM(train = ***M***_AD/NC_).outputScore(***M***_MCI_);
score_PET_ = ELM(train = ***P***_AD/NC_).outputScore(***P***_MCI_);
score_Bio_ = ELM(train = ***B***_AD/NC_).outputScore(***B***_MCI_);
4 scores = [score_MRI_, score_PET,_ score_Bio_]; ## scores∈**R**^N×3^
**Classification and Validation:**
5 for n from 1 to 100:
6 scores = scores[random_permute,:];
**Ten folds cross-validation:**
7 separate scores into ten folds along first dimension;
8 for i from 1 to 10:
testSet = scores[foldth = = i,:];
trainSet = scores[others,:];
record predict = ELM(train = trainSet).classify(testSet);
end for
end for
9 statistics of 100 runs

### Feature Selection With Least Absolute Shrinkage and Selection Operator

Different from other modalities, the MRI features are the morphological characters of all cerebral regions. However, some of them may be aging-related and not AD-related, which can interfere with the classification, and thus need to be excluded. In this study, we adopted LASSO to select only useful MRI features. LASSO is an *L_2_,_1_* norm sparse regression model (Kukreja et al., 2006) and has the following formula:

(1) $$\min_{\alpha }\,0.5\,{||y-D\,\alpha ||}_{2}^{2}+\lambda \,{||\alpha ||}_{1}.\;$$

In formula (1), ***y***∈**R**^1×^*^*N*^* is the vector of *N* labels, and ***D***∈**R***^*N*^*^×^*^*M*^* is a feature matrix that consists of *N* training samples with *M* features in each sample. The variable λ is the penalty coefficient that was set to 0.015 in this study, and **α**∈**R**^1×^*^*M*^* is the target sparse coefficients. When this model is solved, only some coefficients in **α** would be non-zero, where the larger absolute value of these coefficients indicates higher usefulness of the corresponding features. Therefore, the results of **α** can be used to select discriminative features. Unlike previous studies (Lee et al., 2016; Lin et al., 2018), which trained the LASSO model with pMCI/sMCI features for the pMCI/sMCI classification task, we thought the features of AD/NC were more representative and used them to train LASSO model. Then, the features with non-zero coefficients in **α** were selected.

### Extreme Learning Machine

Extreme learning machine is a one-step learning algorithm that is faster and has a higher performance than the support vector machine (Huang et al., 2012; Zeng et al., 2017). There are two types of basic ELM; the first is a feed-forward neural network with only a single layer of randomly generated hidden nodes (Huang et al., 2006). The second type is an ELM with kernels (Huang et al., 2012), which avoids the random generation of an input weight matrix. ELM with kernels yields more stable results and has a higher performance than the feed-forward neural network. In our previous work (Lin et al., 2018), the ELM with kernels showed more efficiency than support vector machine and random forest in the prediction of AD. Therefore, we adopted ELM with a Gaussian kernel in this study. The process of ELM with a Gaussian kernel can be described as follows:

Suppose we have *N* training samples [***x***_1_, ***x***_2_, ⋯, ***x****_*N*_*] and *N* labels. The variable ***x****_*n*_* represents a vector with *M* features of one sample, and ***Y***∈**R***^*N*^*^×2^ is a ground truth label matrix with *N* rows. In each row, the element corresponding to the true label is set to 1, and the other is set to −1. When a new sample, ***x***, is obtained, the label of ***x*** can be predicted as

(2) $$f\,\left(x\right)=\left[\begin{array}{c} \begin{array}{c} K\,\left(x,x_{1}\right)\\ K\,\left(x,x_{2}\right) \end{array}\\ \vdots \\ K\,\left(x,x_{N}\right) \end{array}\right]\,{\left(\Omega +\frac{I}{C}\right)}^{-1}\,Y,$$

where K(***x***, ***x****_*N*_*) is the Gaussian kernel described as

(3) $$K\,\left(u,v\right)=e\,x\,p\,\left(\frac{-{∥u-v∥}^{2}}{\gamma }\right),$$

and **Ω** is an *N* × *N* kernel matrix that is related to the training samples, which is calculated in the training phase as

(4) $$\Omega =\left[\begin{array}{ccc} \begin{array}{c} K\,\left(x_{1},x_{1}\right)\\ \begin{array}{c} K\,\left(x_{2},x_{1}\right)\\ \vdots \end{array}\\ K\,\left(x_{N},x_{1}\right) \end{array} & \ldots & \begin{array}{c} K\,\left(x_{1},x_{N}\right)\\ \begin{array}{c} K\,\left(x_{2},x_{N}\right)\\ \vdots \end{array}\\ K\,\left(x_{N},x_{N}\right) \end{array} \end{array}\right].\;$$

The variable *C* in formula (2) is a regularization coefficient and is set to 1. The variable γ in formula (3) is a parameter of the Gaussian kernel, which is set to 10 times *M* number of features in this study.

The output of formula (2) is a vector with two elements: [*s*_1_, *s*_2_]. When ELM is used as the classifier, the output is the result of comparing the values of *s*_1_ and *s*_2_. In this study, we used the ELM to grade MCI samples, and the output of ELM was modified as *s* = *s*_1_–*s*_2_. When the ELM was trained with AD/NC and tested on MCI, the output score *s* can represent the similarity of MCI-to-AD or NC.

### Classification and Performance Analysis

Ten-fold cross-validation was implemented to assess the performance of the proposed approach. All MCI subjects were separated into 10-folds randomly. In each validation iteration, one different fold was selected as testing data and the other nine folds were used as training data. This process was repeated for 10 iterations. The classification results of 10 iterations were compared to the true labels, and the accuracy, sensitivity, specificity, and area under receiver operating characteristic (ROC) curve (AUC) were calculated. To avoid sampling bias, the 10-fold cross-validation was run 100 times with randomly permuted samples, and the mean and standard deviation of the accuracy, sensitivity, specificity, and AUC were given.

## Experiments and Results

### Results Using Multimodality Data

**FIGURE 2:**
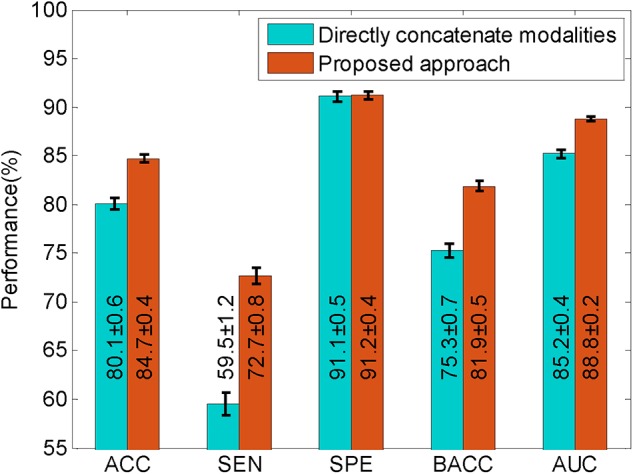
The comparison of the proposed approach with the method that directly concatenates multiple modalities. The black lines superimposed on each bar, and the second number in each bar represents the standard deviations derived from 100 runs of validation. ACC, accuracy; AUC, area under receiver operating characteristic curve; BACC, balanced accuracy; SEN, sensitivity; SPE, specificity.

To evaluate the improvement of the proposed approach, we compared it with the method that directly concatenates multimodal data. The results of the comparison are shown in Figure 2. From these results, we found that the method that directly concatenates the four modalities had a high accuracy and specificity of 80.1 and 91.1%, respectively, but the sensitivity was quite low. For a non-biased performance evaluation, we calculated the balanced accuracy, which is the average of sensitivity and specificity, and obtained 75.3%, which is not optimal. The proposed approach had better results in terms of accuracy and sensitivity, with an accuracy of 84.7% and a sensitivity of 72.7%. This is approximately 13% higher than the direct concatenation method. The proposed approach also has a promising balanced accuracy of 81.9%, which is 6.6% higher than the other method. Beside these scores, we also obtained an improved AUC of 88.8% for our proposed method. This comparison indicates that the proposed approach is more efficient at predicting the MCI-to-AD conversion than the method using directly concatenated multimodal data.

### Contributions of Different Modalities

To reveal the contributions of the different modalities in the proposed method, experiments were conducted with only one modality and one modality absent. The results are listed in Table 2. Because the APOE ϵ4 data failed to classify pMCI/sMCI individually (with balanced accuracy of 55.2%), we used CSF + APOE ϵ4 to demonstrate the effect of APOE ϵ4.

**TABLE 2:** The contributions of different modalities.

**Modalities**	**ACC**	**SEN**	**SPE**	**BACC**	**AUC**
MRI	74.5 ± 0.4%	54.8 ± 0.9%	85.0 ± 0.3%	69.9 ± 0.5%	79.2 ± 0.2%
FDG-PET	76.7 ± 0.4%	55.1 ± 0.8%	88.2 ± 0.5%	71.7 ± 0.4%	80.9 ± 0.2%
CSF	73.0 ± 0.5%	62.5 ± 1.0%	78.7 ± 0.5%	70.6 ± 0.6%	79.0 ± 0.3%
CSF + APOEϵ4	73.9 ± 0.4%	63.2 ± 0.7%	79.7 ± 0.6%	71.4 ± 0.4%	78.8 ± 0.3%
- MRI	81.3 ± 0.5%	67.0 ± 1.0%	89.0 ± 0.5%	78.0 ± 0.6%	86.8 ± 0.2%
- FDG-PET	81.0 ± 0.5%	67.2 ± 0.8%	88.4 ± 0.5%	77.8 ± 0.5%	86.7 ± 0.2%
- CSF	79.6 ± 0.6%	63.5 ± 1.2%	88.3 ± 0.5%	75.9 ± 0.7%	85.8 ± 0.2%
- APOEϵ4	83.2 ± 0.5%	69.8 ± 1.0%	90.4 ± 0.5%	80.1 ± 0.6%	88.7 ± 0.2%
- LASSO	83.5 ± 0.6%	69.0 ± 1.2%	91.2 ± 0.6%	80.1 ± 0.7%	88.8 ± 0.2%
All	84.7 ± 0.4%	72.7 ± 0.8%	91.2 ± 0.4%	81.9 ± 0.5%	88.8 ± 0.2%

*“- modality” means the absence of the modality in experiments. In each cell, the two numbers represent the mean and standard deviation derived from 100 runs of validation. ACC, accuracy; APOE, apolipoprotein E; AUC, area under receiver operating characteristic curve; BACC, balanced accuracy; CSF, cerebrospinal fluid; LASSO, least absolute shrinkage and selection operator; SEN, sensitivity; SPE, specificity.*

**FIGURE 3:**
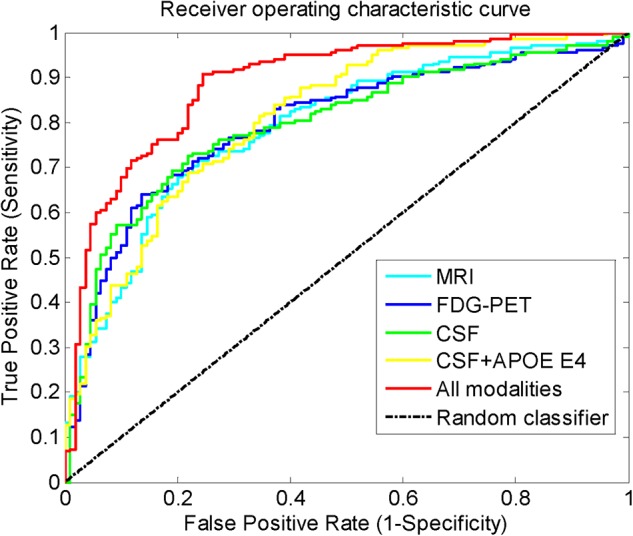
The receiver operating characteristic curves when different modalities were used.

From these results, we can see that when only one modality was used, the performance of the CSF ranked third in terms of accuracy and AUC, but it had the best sensitivity. The APOE ϵ4 feature can slightly improve the results using CSF. FDG-PET achieved the best results, but the best accuracy and balanced accuracy were only 76.7 and 71.7%, respectively. When all modalities were used, the accuracy and balanced accuracy was greatly improved to 84.7 and 81.9%, respectively, and there was also a significant improvement in AUC. Figure 3 shows the improvement in the ROC curves of the proposed approach, when all modalities were used compared with only one modality used. In the situation with one modality absent, it shows that the performance declined without CSF, especially a significant decline of sensitivity, which led to the decline of balanced accuracy. The MRI and FDG-PET had a similar impact on the performance of the proposed method, while the APOE ϵ4 had minimal influence on the performance. Even when all modalities were used, if the LASSO was disabled, the performance suffered from a 1.2 and 1.8% drop in the accuracy and balanced accuracy, respectively, which illustrates the contribution of LASSO.

### Prediction Within Different Periods

**FIGURE 4:**
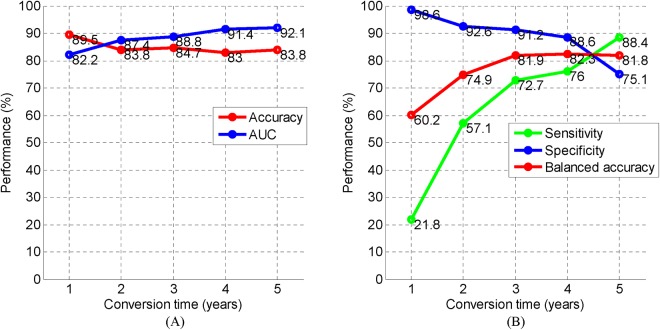
The performance of predicting MCI-to-AD conversion at different time periods. **(A)** Performance of accuracy and AUC. **(B)** Performance of sensitivity, specificity, and balanced accuracy. AD, Alzheimer’s disease; AUC, area under receiver operating characteristic curve; MCI, mild cognitive impairment.

The 3-year cutoff period for predicting MCI-to-AD conversion is not a unique criterion. We also conducted experiments to predict the states of MCI patients with different periods from 1 to 5 years. With the criterion changed, different numbers of pMCI/sMCI for different conversion times were obtained: 46/343 (1 year), 89/268 (2 years), 110/205 (3 years), 119/146 (4 years), 117/62 (5 years). The results of predicting MCI-to-AD conversion at different time periods are shown in Figure 4. From Figure 4A, we can see that the accuracies are all above 83% for 1–5 years prediction. However, from Figure 4B, we found the specificity was high and the sensitivity was low at the point of 1 year, owing to the disparity of the number of individuals with pMCI versus sMCI (46/343), and the balanced accuracy was only 60.2%. At the point of 2 years, the bias is still large: 89/268 pMCI/sMCI. As a result, the balanced accuracy was only 74.9%. At the 3–5-year mark, the bias reduced and the balanced accuracies stabilized at approximately 82%. At the points of 3–5 years, we achieved an accuracy, balanced accuracy, and AUC of 83, 81.8, and 88.8%, respectively. These results show a promising performance of the proposed approach for predicting MCI-to-AD conversion within different periods.

### Experiments on Other Conditions

We also conducted the experiments on different conditions, including:

(i) An Support Vector Machine (SVM) version, in which SVM was the classifier instead of ELM.
(ii) In some studies, neuropsychological test scores (MMSE, clinical dementia rating-sum of boxes, Alzheimer’s disease assessment scale-cognitive subtest, Rey’s auditory verbal learning test, functional activities questionnaire) were included to boost the performance of prediction. Therefore, these neuropsychological test scores were also included and concatenated with grading scores.
(iii) In previous studies (Moradi et al., 2015; Tong et al., 2017a), the definition of sMCI was stricter, and the subjects who converted to AD beyond 3 years or the diagnosis changed from MCI to NC were removed from the sMCI group. Therefore, we also excluded 64 subjects with the same criterion, and then obtained 141 sMCI and 110 pMCI.

The results are listed in Table 3, from which it can be seen that the ELM classifier has a better performance than the SVM classifier. When neuropsychological test scores were included in the proposed method, there was not a significant improvement in accuracy, but the AUC greatly improved. When the ambiguous subjects were excluded from the sMCI group, the performance was further improved. To the best of our knowledge, the accuracy of 87.1% and AUC of 94.7%, achieved in this experiment, are the best for predicting AD.

**TABLE 3:** The experiments on different conditions.

**Classifier**	**Modalities**	**pMCI/sMCI**	**ACC**	**AUC**
SVM	MRI, PET, CSF, APOE	110/205	83.6%	–
ELM	MRI, PET, CSF, APOE	110/205	84.7%	88.8%
ELM	MRI, PET, CSF, APOE, neuropsychological scores	110/205	85.1%	92.6%
ELM	MRI, PET, CSF, APOE, neuropsychological scores	110/141 (ambiguous subjects excluded)	87.1%	94.7%

*ACC, accuracy; APOE, apolipoprotein E; AUC, area under receiver operating characteristic curve; CSF, cerebrospinal fluid; ELM, extreme learning machine; pMCI, progressive mild cognitive impairment; sMCI, stable mild cognitive impairment; SVM, support vector machine.*

## Discussion

In this study, we propose a novel approach for predicting MCI-to-AD conversion with multimodal data. To effectively fuse different modalities and avoid the bias of a number of features in each modality; an ELM-based grading method was employed to calculate a grading score for each modality. The scores of multiple modalities were combined and fed into the ELM classifier to discriminate the pMCI from sMCI. With the help of AD/NC information included in the grading procedure, the scores effectively represented the states of the MCI subjects and were used to predict the AD conversion individually. When the scores from all modalities were combined, the accuracy of prediction was boosted to 84.7%. The results of the experiments conducted on the ADNI cohort demonstrate that: (i) the proposed method with multimodality scores has a much higher accuracy than with a single modality score, such that the proposed method has at least a 10% higher balanced accuracy than when a single modality is used. This means that the complementary information among the multimodal data can be represented by these scores. (ii) Direct concatenation of multimodal data is not the best way of exploiting the complementary information, and the proposed method showed a more efficient fusion of multimodal data and achieved a much better performance. (iii) The proposed method can predict MCI-to-AD conversion of different periods with a high accuracy.

As more modalities bring more complementary information, the performance of the prediction should improve. As shown in Table 3, when the neuropsychological test scores were included in our approach, the AUC improved, but the accuracy only had a 0.4% improvement. The assumption is that there might be an up-boundary for discriminating pMCI from sMCI, from the fact that the diagnosis in ADNI is not 100% reliable (Ranginwala et al., 2008). Therefore, when we defined the sMCI more strictly and excluded ambiguous samples, the accuracy was further boosted to 87.1% as shown in Table 3. It is also observed that the specificity was much higher than sensitivity in Table 2, and we assume the reason for this might be a bias in the number of pMCI against the number of sMCI. This can be explained in Figure 4B that shows that as the bias in the number of pMCI versus sMCI decreased, a similar specificity and sensitivity were obtained.

Although the proposed approach achieved a promising result in predicting AD conversion, it requires four modalities, which is difficult to obtain in clinical practice. However, in the research of longitudinal regression for modeling the trajectory of AD progression, it is crucial to estimate the cognitive states of patients. In our future work, we will consider the use of the ELM-based grading method proposed in this study to improve the accuracy of longitudinal regression for AD trajectory modeling.

In the proposed approach, the feature selection was only applied to MRI features since the PET features were from five AD-related regions and the three CSF biomarkers and APOE ϵ4 gene contained useful information about AD. Because the MRI features from the FreeSurfer analysis were morphology features of whole brain, inevitably it had to include some useless features. As a result, LASSO was employed to do the feature selection on MRI features, and it improved the results of prediction. To explore which MRI features were selected, we have listed the top 10 features in Table 4. We can observe that the volumes and thicknesses of the hippocampus, amygdala, temporal lobe, and entorhinal cortex play an important role in the detection of AD, which is consistent with previous studies (Van Hoesen et al., 1991; Convit et al., 2000; Mu and Gage, 2011; Poulin et al., 2011).

**TABLE 4:** The top 10 AD-related MRI features from LASSO feature selection.

**Num.**	**MRI features**
1	Volume of left hippocampus
2	Volume of left amygdala
3	Volume of left inferior lateral ventricle
4	Surface area of left isthmus cingulate
5	Volume of right hippocampus
6	Volume of left inferior temporal
7	Cortical thickness average of left middle temporal
8	Cortical thickness standard deviation of right transverse temporal
9	Cortical thickness standard deviation of right lateral orbitofrontal
10	Cortical thickness average of right entorhinal

*AD, Alzheimer’s disease; LASSO, least absolute shrinkage and selection operator.*

## Conclusion

In this study, we have developed an ELM-based grading method to fuse multimodal data for the prediction of MCI-to-AD conversion within 3 years. With the input of four modalities: MRI, FDG-PET, CSF, and gene presence, we achieved a promising result with an accuracy of 84.7% and AUC of 88.8%. When compared with method that directly concatenates multiple modalities, the proposed approach outperformed the other in terms of accuracy and AUC. The experiments demonstrated that this approach can also predict AD conversion of other periods with a similar performance of the 3-year prediction.

## Data Availability Statement

The datasets generated for this study are available on request to the corresponding author.

## Ethics Statement

As per ADNI protocols, all procedures performed in studies involving human participants were in accordance with the ethical standards of the institutional and/or national research committee and with the 1964 Helsinki declaration and its later amendments or comparable ethical standards. The ADNI data collection was carried out after obtaining written informed consent from the participants. More details can be found at adni.loni.usc.edu.

## Author Contributions

WL and TT conceived the study, designed the experiments, analyzed the data, and wrote the manuscript. QG provided the preprocessed data. JY and ZC carried out experiments. CF and WC helped to analyze the data and experiments result. MD and TT revised the manuscript.

## Conflict of Interest

The authors declare that the research was conducted in the absence of any commercial or financial relationships that could be construed as a potential conflict of interest.

## References

[B1] Cheng B. Liu M. Zhang D. Munsell B. C. Shen D. (2015). Domain transfer learning for MCI conversion prediction. *IEEE Trans. Biomed. Eng.* 62 1805–1817. 10.1109/TBME.2015.2404809 25751861 PMC4474791

[B2] Convit A. De Asis J. De Leon M. Tarshish C. De Santi S. Rusinek H. (2000). Atrophy of the medial occipitotemporal, inferior, and middle temporal gyri in non-demented elderly predict decline to Alzheimer’s disease. *Neurobiol. Aging* 21 19–26. 10.1016/s0197-4580(99)00107-4 10794844

[B3] Coupé P. Eskildsen S. F. Manjón J. V. Fonov V. S. Collins D. L. (2012). Simultaneous segmentation and grading of anatomical structures for patient’s classification: application to Alzheimer’s disease. *Neuroimage* 59 3736–3747. 10.1016/j.neuroimage.2011.10.080 22094645

[B4] Eskildsen S. F. Coupé P. García-Lorenzo D. Fonov V. Pruessner J. C. Collins D. L. (2013). Prediction of Alzheimer’s disease in subjects with mild cognitive impairment from the ADNI cohort using patterns of cortical thinning. *Neuroimage* 65 511–521. 10.1016/j.neuroimage.2012.09.058 23036450 PMC4237400

[B5] Fischl B. Dale A. M. (2000). Measuring the thickness of the human Cereb. Cortex from magnetic resonance images. *Proc. Natl. Acad. Sci. U.S.A.* 97 11050–11055. 10.1073/pnas.200033797 10984517 PMC27146

[B6] Fischl B. van der Kouwe A. Destrieux C. Halgren E. Segonne F. Salat D. H. (2004). Automatically parcellating the human Cereb. *Cortex Cereb. Cortex* 14 11–22. 10.1093/cercor/bhg087 14654453

[B7] Gray K. R. Aljabar P. Heckemann R. A. Hammers A. Rueckert D. (2013). Random forest-based similarity measures for multi-modal classification of Alzheimer’s disease. *Neuroimage* 65 167–175. 10.1016/j.neuroimage.2012.09.065 23041336 PMC3516432

[B8] Hamel R. Kohler S. Sistermans N. Koene T. Pijnenburg Y. van der Flier W. (2015). The trajectory of cognitive decline in the pre-dementia phase in memory clinic visitors: findings from the 4C-MCI study. *Psychol. Med.* 45 1509–1519. 10.1017/S0033291714002645 25407094

[B9] Hinrichs C. Singh V. Xu G. Johnson S. C. (2011). Predictive markers for AD in a multi-modality framework: an analysis of MCI progression in the ADNI population. *Neuroimage* 55 574–589. 10.1016/j.neuroimage.2010.10.081 21146621 PMC3035743

[B10] Hu T. K. Lin Y. Y. Hsiu P. C. (2018). “Learning adaptive hidden layers for mobile gesture recognition,” in *Thirty-Second AAAI Conference on Artificial Intelligence* (Palo Alto, CA: AAAI Press).

[B11] Huang G. B. Zhou H. Ding X. Zhang R. (2012). Extreme learning machine for regression and multiclass classification. *IEEE Trans. Syst. Man Cybern.* 42 513–529. 10.1109/TSMCB.2011.2168604 21984515

[B12] Huang G. B. Zhu Q. Y. Siew C. K. (2006). Extreme learning machine: theory and applications. *Neurocomputing* 70 489–501. 10.1016/j.neucom.2005.12.126

[B13] Iaccarino L. Chiotis K. Alongi P. Almkvist O. Wall A. Cerami C. (2017). A cross-validation of FDG- and amyloid-PET biomarkers in mild cognitive impairment for the risk prediction to dementia due to Alzheimer’s disease in a clinical setting. *J. Alzheimers Dis.* 59 603–614. 10.3233/JAD-170158 28671117

[B14] Jack J. C. Jr. Knopman D. S. Jagust W. J. Shaw L. M. Aisen P. S. Weiner M. W. (2010). Hypothetical model of dynamic biomarkers of the Alzheimer’s pathological cascade. *Lancet Neurol.* 9 4–5. 20083042 10.1016/S1474-4422(09)70299-6 PMC2819840

[B15] Kim J. Lee B. (2018). Identification of Alzheimer’s disease and mild cognitive impairment using multimodal sparse hierarchical extreme learning machine. *Hum. Brain Mapp.* 39 3728–3741. 10.1002/hbm.24207 29736986 PMC6866602

[B16] Kohannim O. Hua X. Hibar D. P. Lee S. Thompson P. M. (2010). Boosting power for clinical trials using classifiers based on multiple biomarkers. *Neurobiol. Aging* 31 1429–1442. 10.1016/j.neurobiolaging.2010.04.022 20541286 PMC2903199

[B17] Kukreja S. L. Löfberg J. Brenner M. J. (2006). A least absolute shrinkage and selection operator (LASSO) for nonlinear system identification. *IFAC Proc.* 39 814–819. 10.3182/20060329-3-au-2901.00128

[B18] Lambert J. C. Ibrahimverbaas C. A. Harold D. Naj A. C. Sims R. Bellenguez C. (2013). Meta-analysis of 74,046 individuals identifies 11 new susceptibility loci for Alzheimer’s disease. *Alzheimers Demen.* 9 1452–1458. 10.1038/ng.2802 24162737 PMC3896259

[B19] Landau S. M. Harvey D. Madison C. M. Koeppe R. A. Reiman E. M. Foster N. L. (2011). Associations between cognitive, functional, and FDG-PET measures of decline in AD and MCI. *Neurobiol. Aging* 32 1207–1218. 10.1016/j.neurobiolaging.2009.07.002 19660834 PMC2891865

[B20] Landau S. M. Harvey D. Madison C. M. Reiman E. M. Foster N. L. Aisen P. S. (2010). Comparing predictors of conversion and decline in mild cognitive impairment. *Neurology* 75 230–238. 10.1212/WNL.0b013e3181e8e8b8 20592257 PMC2906178

[B21] Lee S. H. Bachman A. H. Yu D. Lim J. Ardekani B. A. Alzheimer’s Disease, (2016). Predicting progression from mild cognitive impairment to Alzheimer’s disease using longitudinal callosal atrophy. *Alzheimers Dement.* 2 68–74. 10.1016/j.dadm.2016.01.003 27239537 PMC4879655

[B22] Lei B. Siping C. Dong N. Tianfu W. (2016). Discriminative learning for Alzheimer’s disease diagnosis via canonical correlation analysis and multimodal fusion. *Front. Aging Neurosci.* 8:77. 10.3389/fnagi.2016.00077 27242506 PMC4868852

[B23] Lin W. Tong T. Gao Q. Guo D. Du X. Yang Y. (2018). Convolutional neural networks-based MRI image analysis for the Alzheimer’s disease prediction from mild cognitive impairment. *Front. Neurosci.* 12:777 10.3389/fnins.2018.00777 PMC6231297 30455622

[B24] Liu M. Cheng D. Wang K. Wang Y. Alzheimer’s Disease Neuroimaging Initiative, (2018). Multi-modality cascaded convolutional neural networks for Alzheimer’s disease diagnosis. *Neuroinformatics* 16 295–308. 10.1007/s12021-018-9370-4 29572601

[B25] Liu S. Liu S. Cai W. Che H. Pujol S. Kikinis R. (2015). Multimodal neuroimaging feature learning for multiclass diagnosis of Alzheimer’s disease. *IEEE Trans. Biomed. Eng.* 62 1132–1140. 10.1109/tbme.2014.2372011 25423647 PMC4394860

[B26] Lu D. Popuri K. Ding G. W. Balachandar R. Beg M. F. Alzheimer’s Disease Neuroimaging Initiative, (2018). Multiscale deep neural network based analysis of FDG-PET images for the early diagnosis of Alzheimer’s disease. *Med. Image Anal.* 46 26–34. 10.1016/j.media.2018.02.002 29502031

[B27] Markesbery W. R. Lovell M. A. (2010). Neuropathologic alterations in mild cognitive impairment: a review. *J. Alzheimers Dis.* 19 221–228. 10.3233/jad-2010-1220 20061641 PMC2872776

[B28] Moradi E. Pepe A. Gaser C. Huttunen H. Tohka J. Alzheimer’s Disease, (2015). Machine learning framework for early MRI-based Alzheimer’s conversion prediction in MCI subjects. *Neuroimage* 104 398–412. 10.1016/j.neuroimage.2014.10.002 25312773 PMC5957071

[B29] Mosconi L. Mistur R. Switalski R. Tsui W. H. Glodzik L. Li Y. (2009). FDG-PET changes in brain glucose metabolism from normal cognition to pathologically verified Alzheimer’s disease. *Eur. J. Nucl. Med. Mol. Imaging* 36 811–822. 10.1007/s00259-008-1039-z 19142633 PMC2774795

[B30] Mu Y. Gage F. H. (2011). Adult hippocampal neurogenesis and its role in Alzheimer’s disease. *Mol. Neurodegener.* 6:85. 10.1186/1750-1326-6-85 22192775 PMC3261815

[B31] Niemantsverdriet E. Valckx S. Bjerke M. Engelborghs S. (2017). Alzheimer’s disease CSF biomarkers: clinical indications and rational use. *Acta Neurol. Belg.* 117 591–602. 10.1007/s13760-017-0816-5 28752420 PMC5565643

[B32] Oliveira P. P. D. M. Nitrini R. Busatto G. Buchpiguel C. Amaro E. (2010). Use of SVM methods with surface-based cortical and volumetric subcortical measurements to detect Alzheimer’s disease. *J. Alzheimers Dis.* 19 1263–1272. 10.3233/JAD-2010-1322 20061613

[B33] Poulin S. P. Dautoff R. Morris J. C. Barrett L. F. Dickerson B. C. Alzheimer’s Disease Neuroimaging Initiative, (2011). Amygdala atrophy is prominent in early Alzheimer’s disease and relates to symptom severity. *Psychiat. Res. Neuroim.* 194 7–13. 10.1016/j.pscychresns.2011.06.014 21920712 PMC3185127

[B34] Querbes O. Aubry F. Pariente J. Lotterie J.-A. Demonet J.-F. Duret V. (2009). Early diagnosis of Alzheimer’s disease using cortical thickness: impact of cognitive reserve. *Brain* 132 2036–2047. 10.1093/brain/awp105 19439419 PMC2714060

[B35] Ranginwala N. A. Hynan L. S. Weiner M. F. White C. L. III (2008). Clinical criteria for the diagnosis of Alzheimer disease: still good after all these years. *Am. J. Geriat. Psychiat.* 16 384–388. 10.1097/JGP.0b013e3181629971 18448850

[B36] Suk H.-I. Lee S.-W. Shen D. Alzheimer’s Disease Neuroimaging Initiative, (2014). Hierarchical feature representation and multimodal fusion with deep learning for AD/MCI diagnosis. *NeuroImage* 101 569–582. 10.1016/j.neuroimage.2014.06.077 25042445 PMC4165842

[B37] Tong T. Gao Q. Guerrero R. Ledig C. Chen L. Rueckert D. (2017a). A novel grading biomarker for the prediction of conversion from mild cognitive impairment to Alzheimer’s disease. *IEEE Trans. Biomed. Eng.* 64 155–165. 10.1109/TBME.2016.2549363 27046891

[B38] Tong T. Gray K. Gao Q. Chen L. Rueckert D. The Alzheimer’s Disease, (2017b). Multi-modal classification of Alzheimer’s disease using nonlinear graph fusion. *Pattern Recogn.* 63 171–181. 10.1016/j.patcog.2016.10.009

[B39] Van Hoesen G. W. Hyman B. T. Damasio A. R. (1991). Entorhinal cortex pathology in Alzheimer’s disease. *Hippocampus* 1 1–8. 10.1002/hipo.450010102 1669339

[B40] Vos T. Allen C. Arora M. Barber R. M. Bhutta Z. A. Brown A. (2016). Global, regional, and national incidence, prevalence, and years lived with disability for 310 diseases and injuries, 1990–2015: a systematic analysis for the global burden of disease study 2015. *Lancet* 388 1545–1602. 27733282 10.1016/S0140-6736(16)31678-6 PMC5055577

[B41] Vounou M. Janousova E. Wolz R. Stein J. L. Thompson P. M. Rueckert D. (2012). Sparse reduced-rank regression detects genetic associations with voxel-wise longitudinal phenotypes in Alzheimer’s disease. *Neuroimage* 60 700–716. 10.1016/j.neuroimage.2011.12.029 22209813 PMC3551466

[B42] Walhovd K. B. Fjell A. M. Brewer J. McEvoy L. K. Fennema-Notestine C. Hagler D. J. (2010). Combining MR imaging, positron-emission tomography, and CSF biomarkers in the diagnosis and prognosis of Alzheimer disease. *Am. J. Neuroradiol.* 31 347–354. 10.3174/ajnr.A1809 20075088 PMC2821467

[B43] Westman E. Muehlboeck J.-S. Simmons A. (2012). Combining MRI and CSF measures for classification of Alzheimer’s disease and prediction of mild cognitive impairment conversion. *Neuroimage* 62 229–238. 10.1016/j.neuroimage.2012.04.056 22580170

[B44] Young J. Modat M. Cardoso M. J. Mendelson A. Cash D. Ourselin S. (2013). Accurate multimodal probabilistic prediction of conversion to Alzheimer’s disease in patients with mild cognitive impairment. *Neuroimage Clin.* 2 735–745. 10.1016/j.nicl.2013.05.004 24179825 PMC3777690

[B45] Zeng N. Zhang H. Liu W. Liang J. Alsaadi F. E. (2017). A switching delayed PSO optimized extreme learning machine for short-term load forecasting. *Neurocomputing* 240 175–182. 10.1016/j.neucom.2017.01.090

[B46] Zhang D. Wang Y. Zhou L. Yuan H. Shen D. (2011). Multimodal classification of Alzheimer’s disease and mild cognitive impairment. *Neuroimage* 55 856–867. 10.1016/j.neuroimage.2011.01.008 21236349 PMC3057360

